# Selections and Abstracts

**Published:** 1913-04

**Authors:** 


### SELECTIONS AND ABSTRACTS

### INSTRUCTION IN HYGIENE IN MEDICAL COLLEGES AND THE TRAINING OF HEALTH OFFICERS*

F. F. Wesbrook, M. D., Minneapolis

*Professor of Pathology and Bacteriology and Dean, College of Medicine and Surgery, University of Minnesota; President-Elect, University of British Columbia*

In America we have been slow to recognize the need of special training for the various branches of public service. Many examples lie close at hand We are proud to show visitors that our most imposing and best buildings are for the training of our children, those who are to carry on the work after we have laid it down. Yet we entrust the training of our girls and boys to those who are school teachers pro tempore and whose ultimate graduation at the altar, at the bar, in the profession of medicine or in business being constantly in mind, is apt to lower pedagogic efficiency. Corporation lawyers often use the bench as a postgraduate training. The interests of the corporations demand and receive the services of the best and most highly paid talent, while on the other hand, the interests of the public are too often left to the individual who needs the opportunity either as a stepping-stone to something higher, or because he finds himself unfitted for the practice of law.

Our health officers are frequently given to the incoming recent graduate, from a mixture of motives. It is freely admitted by the older practictioners that service as a health officer is likely to make the individual so unpopular that he will move on. The newcomer is therefore cajoled into the position. Frequently the older medical men have too much to lose from their private practices to be able to afford the questionable luxury of public service. Occasionally, too, the position of health officer, or superintendent of hospital or other institution is regarded by the medical profession as a suitable gift for

*Presented before the Section on State and Municipal Hygiene at the International Congress on Hygiene and Demography, Washington, D. C., September 1912.

the man who has shown inaptitude or encountered misfortune in private practice. Too often, the position of city engineer is given to the land surveyor or other individual who needs it, but whose original training and field practice have not been such as to fit him for the care of this phase of the public interest.

On every hand, we seek through volunteer agencies to secure satisfactory administration of laws which should become operative through official mechanisms, thus possibly postponing the final solution of our difficulties. Thereby, we add tremendous burdens to those who volunteer for public service and work them at great disadvantage, because frequently their main qualification is interest rather than detailed knowledge.

We need not dwell, however, on what has been. We can better discuss what is to be. Without delaying further to inquire into the mixture of causes or pausing to fix the blame for the apathy of the public in regard to its own welfare, we recognize the need for radical reform. Here in America, we who are optimistically inclined believe that the time has come when a demand is to be made in increasing degree for trained specialists, who shall devote their whole time and energy to the various phases of public health protection. We must, however, adjust the pay, as well as the authority, of our health officials and workers to the responsibilities which the public expects them to assume. We must guarantee them tenure of office dependent alone on efficiency. We must continue to insist that they be trained before they assume office and not expect them to gain their experience at the expense of the public.

The present great wave of publicity cannot but develop a public and private discrimination which will demand efficiency. Will the people who demand the efficient service be willing to pay the price for efficiency?

Altruistic and not financial considerations should dominate our discussion of the public welfare. It is useless, however, to expect men and women to spend thousands of dollars in preparation for life's work, unless they may look forward to a fair return upon the invested capital. There is a yet more important investment of which we sometimes lose sight. I refer to the years of youth and enthusiasm which cannot later be replenished as can an exhausted exchequer. On such a combined investment of the individual, it is just that the public should make a return in recognition and the wherewithal to live.

What workers are needed in public health fields is a question we must ask ourselves in attempting to provide for their training. The answer to this is that experts in every line who approach social and economic activities from the health standpoint are even now required. All of us are naturally solicitous about the future and desirous that better training be provided for our successors. Complexities in increasing degree are, however, inevitable, notwithstanding the rapid advance in man's knowledge concerning his own environment. This very advance demands greater specialization and therefore means increased difficulty of corordination. Furthermore, increase in density of population and in facilities for transportation very naturally increase our difficulties in the adjustment of the duties and responsibilities of individuals to each other and of the individual to the mass.

In the ultimate analysis, no offcial public health protective mechanism can operate without the intelligent interest and the sympathetic support of the medical profession, since its members are responsible for individual and family health, which in the mass represents public health. No attempt should be made, however, to make sanitary experts out of physicians by the ordinary medical training. They should have sufficient foundation to enable them to meet their own responsibilities in general or special lines and to articulate with the official health agencies.

No nation has more rapidly advanced in methods, machinery and requirements for the teaching of medicine than has the United States, and the training now given to undergraduates in our best colleges, is not excelled by that of any other country. They require from six to eight years in college or university, medical school and hospital. This provides a general training in medicine but does not quality graduates as specialists in private practice or as leaders in public medicine.

Engineering colleges, notable the Massachusetts Institute of Technology, are providing courses in sanitary and municipal engineering which require a four or five years' training on the completion of which graduates are incompetent to assume important responsible public health positions without further training in relation to their "disease point of view." In our leading colleges and universities in conection with the departments of economics and sociology, training is provided for social workers and others who have important public duties to perform. They need, however, some more definite ideas concerning structural problems and the engineer's point of view, as well as of basic biologic, chemical and physical principles and modern conceptions and methods of dealing with health problems. The recently aroused interest in the study of municipal problems is important, if it does not leave out of consideration or subordinate state, federal and international relations, as well as that most urgent of our problems, namely, rural betterment, which has so many sanitary complications.

Health departments of the future and other official and volunteer agencies for promoting public health must secure the coordinated service of various groups of physicians trained in many diverse lines; of economists, of social workers, of statisticians, of engineers of various trainings and ambitions, of dentists, of hospital superintendents, of bacteriologists, pathologists, and chemists, of meat, milk and food inspectors, of visiting nurses, school inspectors, physical trainers, inspectors of industries, teachers of public and personal hygiene; and legislators, lawyers and even policemen must be impressed into the service.

Now many of these workers require special training in advance in relation to their public duties. How should this training be provided and who shall teach?

Universities with the necessary departments must cooperate with the various federal, state or municipal official and volunteer public-health agencies. The state universities of the middle west can perhaps more easily make the needed articulations with other interests and thus obtain opportunities for practical instruction, than can the privately endowed institutions. If these universities are fortunate enough to be located, like the University of Minnesota, in progressive cities, the various phases of municipal hygiene may be studied practically, and more efficient and practical public-health teaching "clinics" conducted.

### An Outline of Cooperative Work

In laying out a course for Minnesota, the following agencies, among others, have been considered in plans for cooperative work and should be expected to furnish teachers and opportunities for practical observation, with actual field work in the case of certain of them because of their need of trained workers.

1. The University of Minnesota, a state institution.

A. The colleges of medicine, denistry and pharmacy and train teachers and become more productive in research.

B. The College of Agriculture in relation to rural sanitary betterment, food production and other such problems has much in the way of opportunity and responsibilty.

C. The College of Engineering in matters involving construction and expenditure of funds, particularly in relation to water, wastes, ventilation, building construction, etc., should continue and extend its work in sanitation and public health.

D. The School of Chemistry should continue to participate in sanitary research and public-health teaching.

E. The College of Science, Literature and the Arts in its scientific laboratories of biology, physics and phychology and its departments of economics and sociology is in a position to contribute and receive much of very real value.

F. The College of Education, particularly in medical school inspection and the teaching of personal hygiene and physical training for children, has very real needs and offers unsurpassed opportunity for coordinating effort.

G. The University Department of Physical Training should be extended and built into a health-promoting and teaching organization.

H. The University health officer, when such a mechanism is provided will furnish the means for public-health teaching and research in the discharge of his daily work which is the School for Nurses must continue to graduate practitioners, so much needed.

I. The University Extension Bureau can undertake no more practical and important work than the training of experts in health publicity and the spread of the gospel of health.

J. The College of Law and other departments and mechanisms of the university can all help and be helped by such affiliations.

2. The State Board of Health in its various branches, including administrative, engineering, epidemiologic, statistical and laboratory work, is in need of trained workers and should be glad to help in its many fields for practical training because it thereby helps itself. Three of the four divisions are already housed by the state university and cooperative arrangements should be still further extended. One of its division heads is on the university teaching staff in medicine and one in sanitary engineering. Both the engineering and medical graduates are therefore better prepared to meet their public responsibilities and to be of real help to the State Board of Health and their own local health boards.

3. The state departments of education, as also the city departments of education of St. Paul and Minneapolis, have many problems educational and sanitary which cooperative effort alone can solve.

4. The board of control institutions, which include state hospitals for the insane, schools for the feeble-minded, deaf and blind; state institutions for tuberculosis; schools for dependent and neglected children; correctional institutions, etc., are increasingly embarrassed by lack of trained workers. They will increase the efficiency of their own staffs by the stimulus of having them assist in giving instruction.

5. The State Live Stock Sanitary Board has many problems to solve and is helping to solve those which affect human health. It is already cooperating with the University Agricultural College.

6. The state dairy and food department and the federal meat inspection service at South St. Paul are important instruments not only in the protection of human welfare but in the teaching of public sanitation.

7. The state attorney-general's office can help in the study of the legal mechanism for the control of disease and in the teaching of those who are to become expert in the use of legal and other tools for protecting health interests. The university should also prove useful to that arm of the state if it could furnish medical and other scientific experts for the court work of the state and develop an "institute of forensic medicine."

8. The state labor bureau has much both to give and receive in such a combined effort.

9. The municipal health departments of Minneapolis and St. Paul, the city engineering departments in relation to water supplies, sewerage disposal, etc., the municipal hospitals for contagious and other diseases, and the municipal health agencies of smaller cities are important to the success of such an organization.

10. The Associated Charities organization of Minneapolis and St. Paul need workers and can teach much.

11. The State Association for the Prevention and Relief of Tuberculosis and other voluntary organizations and agencies should all present their work and activities to the students of public health and would undoubtedly welcome the advent of any mehanism for training workers

The list given above is one which has already been presented* to the Minnesota State Sanitary Conference in 1910, to which I have made some additions which should doubtless be extended still further.

---

*Wesbrook, F. F.: Diplomas in Public Health, Jour. Minnesota State Med. Assn., 1910, N. S., xxx, 504.

Time does not permit of a detailed outline of such courses but by interesting such agencies and workers as those mentioned, and by efficient instruction at our state university, and at certain stages of the teaching by placing the students in the field with those actually engaged in the work, an ideal training could be provided.

In regard to the teachers, it is not enough that a single individual should give all the instruction. A lasting impression concerning the responsibilities involved in daily practice in a particular sanitary field can be had only from intimate contact with those who are specialists in that line. The different workers, factors and fields involved should be presented to the student so that he may realize more clearly what is expected of him in his private and public capacities.

Public health work constitutes a series, and it should be a coordinated series, of specialties. For its successful teaching, therefore, a large staff is required, just as a large staff of medical teachers is required to teach modern medicine in all its phases. It seems probable that the development, even with those universities which are able to furnish the facilities already mentionel and to secure the proper articulation, will first be along the lines of departments of pubilc health, and later, replacement of these by schools or colleges of public health, having on their faculties many members of the staffs of other colleges in the university; and in addition, lecturers from various state, municipal and federal departments, bureaus and institutions, and officers and workers of voluntary associations and certain private institutions.

### Curriculum

Little has been said about curriculum, which must include the following, among other topics:

1. Legal mechanisms, federal, state and municipal, for the control of disease.

2. Vital statistics, or the bookkeeping of public health.

3. Consideration of the transmissible diseases and their means of spread and of prevention, whereby the nature of the virus, or the microbe, or exciting cause, is considered; the atrium or gateway through which the virus gains entrance into the body; the distribution of the exciting cause in the infected individual; methods of its elimination; its point of exit; the role played by intermediate carriers or vehicles and the modifications of procedure which are involved by our possession of any specific means of protection based on the use of vaccines, antitoxins, or other such agents.

4. Disinfection.

Occupational diseases, exclusive of infection.

6. Milk-supply in relation to disease.

7. Food supply; meat inspection, etc.

8. Water-supply and sewerage disposal.

9. Sanitary engineering and architecture, including plumbing, ventilation, hospital, school and other public building sanitation as well as housing conditions.

10. Social economics and the economic cost of disease; saving to be effected through conservation; governmental and other life and sickness insurance mechanisms.

11. Publicity in relation to public health.

12. Sanitation of travel.

13. Sanitary aspects of embalming, funeral direction, transportation of the dead.

14. Inspection of hotels and restaurants in relation to cleanliness, sanitary facilities and also medical inspection of those who handle food, water, etc.

15. School hygiene in its many phases.

16 The hygiene and the conduct of public institutions for the insane, feeble-minded, deaf, blind and also our correctional institutions.

17. Eugenics in its educational and legal aspects.

18. Infant welfare.

19. The hygiene of venereal diseases.

20. Mental hygiene.

21. Naval and military hygiene.

22. Tropical medicine.

23. Personal hygiene, to include nutrition, instruction on food values and preparation, exercise, clothing, individual habits of work, sleep, etc.; sex hygiene.

24. Alcoholism in its personal and public phases.

25. Dental and oral prophylaxis considered in relation both to personal hygiene and to public teaching and practice.

It is not practical that the instruction given to various types of public health workers should be identical, in regard to the length of the course, the subjects included, or the amount and character of instruction in each course. It is not necessary or even desirable that students should all have the initial training. It seems fairly certain in fact, that very different courses will be demanded and its therefore only logical that different degrees or diplomas should be granted, according to the nature and length of the training which has been received and the character of the public-health service for which the individual seeks to qualify. For those who expect to serve as medical officers of health, shall we demand a preliminary graduation in medicine? If so, shall we give a three years' training in the public health school? This question can be answered best by saying that thus far, the American universities which have developed such courses have had practically no students. It is absolutely necesasry that all workers in the public-health field should acquire the disease point of view, but it does not seem feasible or desirable that of every worker in public health, a medical degree should be demanded. Physicians who go into public health work find that to their knowledge of medicine must be added many other important items of experience. Engineers are ready to make the same admissions about their deficiencies of training. Sociologists and economists have already found that it is impossible for one individual or department to specialize in every phase of human activity and relationship. They realize that unless due care is exercised, they are likely to lack discrimination in relation to medical matters just as the engineers accuse the medical men of being deficient in quantitative sense. We shall not arrive at our destination however, if we stop when we have pointed out each other's faults. Only by team work in which each recognizes the importance and strength as well as the needs of his collaborators shall we advance.

As an additional safeguard of our public health, if it seems desirable, we may require license of the trained worker, in addition to his diploma or certificate.

The additional duty might be put on our State Board of Health of examining for license those who seek to enter any of the sanitary fields, for which they have received special training as evidenced by their diplomas or certificates.

The Danish plan of a provisional appointment to public office for a limited period, say for six months, in order to determine practical fitness, before the appointment is confirmed, has very decided advantages. This proceeding is perhaps more applicable, however, to the position of medical officer of health than it is to some of the subordinate positions.

For the training of public-health workers, not only is it necessary that some knowledge of man's structure and the functions of his various organs be learned, but his relation to his environment must be understood. The practical consideration of costs and of structural detail must not be lost sight of since much of our program must needs be constructive. Above all things a knowledge of mankind is quite as essential as a knowledge of man. Human nature will continue to be just as important a factor in the future as in the past. Men and women of exceptional caliber and of great breadth and depth of training are needed and will be forthcoming just as rapidly as the public realizes its need. Our problem then is alrgely that of developing public interest and support by the rationality of our methods of work, and our recommendations for the betterment of existing means.—*Journal A. M. A.*

---

### AIDING THE PROTECTIVE PROCESSES OF THE BODY

The investigations which have been made during the last thirty years as to the means by which the body protects itself from infectious diseases have revealed the fact that these means are very numerous and do not act along one single line. In some instances, phagocytosis seems to be the chief factor which the body relies upon in combating infection. In other instances bacteriologic substances in the serum may possibly be the chief agent. In others, antidotal or antitoxic substances are produced. In still others, by the development of fever the tissues of the individual infected are rendered a less favorable site for the growth a less favorable site for the growth of a microorganism, or they are able by increased oxidation processes to more readily burn up or destroy poisons which are originated directly or indirectly by invading germs. By the use of antitoxin we provide the body with a foreign antidote to the poison produced by diphtheria or tetanus, and while it is true that diphtheria toxin will pass through a porcelain filter before being mixed with antitoxin and fails to pass or passes with great difficulty through such a filter after it is so mixed, it is nevertheless a fact that neither the chemist nor the biologist can tell us exactly what toxin and antitoxin are.

When Wright brought forward his fundamental and original views ocncerning so-called vaccine or opsonic therapy, he led us to believe that in this line would be developed the most important therapeutic advance of the century. Vaccine therapy has taken, in all probability, a permanent place as a means of combating certain diseases, but there can be no doubt that for some reason, as yet to be discovered, it signally fails in quite a large proportion of cases in which, so far as our knowledge goes, we have every theoretical right to believe it will succeed. The practical impossibility of studying the opsonic index by the general practitioner, who by this means might determine the best type of cases for vaccine therapy, heavily handicaps this line of treatment. Not only is it impossible for the average man to reach accurate conclusions as to the opsonic index, but even those who have worked with it constantly and persistently arrive at such diverse results that all but the most enthusiastic have given up the task as almost hopeless. One point in regard to so-called vaccine therapy which seems to be well assured is that it cannot be of advantage unless the body still possess sufficient vitality to be capable of being aroused into still greater resistance to the invading organism. In other words, if the powers of resistance are so diminished that they are beyond stimulation vaccine therapy must inevitably fail and perhaps do harm; whereas if, to use a somewhat homely phrase, the powers of resistance are torpid or lazy, the use of vaccine therapy may spur them into sufficient activity to win the battle with the invading microorganism. Another cause for the failure of vaccine therapy is the almost absolute necessity of employing a dead micro-organism identical with the living one which is responsible for the disease. The difficulty in obtaining a pure culture and in making an autogenous vaccine has, therefore, led to the employment of vaccines which are not autogenous in the hope that the right one might be hit upon. In other words, a very distinct and well-defined chance is taken by the practitioner who uses an autogenous vaccine, and a still greater chance by one who employs a vaccine which is not autogenous. In the latter case it is almost like the toss of a penny in determining whether good is to result or whether at least no advantage is to be gained.

In some instances, vaccines fail because the symptoms which are present are not induced by a single germ but by associated germs, each one of which is responsible for at least one color in the picture of disease, and recognizing this fact physicians often employ what is known as a polyvalent, combined or mixed vaccine. Here, again, the treatment must be largely in the nature of an experiment, since they have no means of determining whether a single or a large number of different organisms are responsible. Furthermore, they cannot tell which organism is present in so large a majority that it is chiefly responsible for the conditions manifested. Nevertheless, these polyvalent vaccines are largely employed, and there is every reason to believe that their continued use depends upon the fact that, in some cases at least, the results which follow their employment are advantageous.

When several hundred million dead bacteria are injected into the body, no one supposes that it is the dead bacteria themselves which produce the therapeutic effect, but rather substances which these dead bacteria contain. In other words, the microorganisms contain their own particular quantity of toxic material, and this toxic material being in moderate amount in a total injection spurs the protective processes of the body to increased activity.

About eighteen months ago the Therapeutic Gazette published an article by Dr. Schafer in which he advocated the employment of what is practically an extract or filtrate of the substances contained in pathogenic micro-organisms. He did not advocate the employment of any one strain of organism, but rather that the educts of a number of strains should be mixed together and used in this manner, the idea being that practically all infections are associated infections, and that while one organism may predominate, it is usually associated with a number of others in sufficient proportions to make them influential factors in the case. We did not publish this article until we had presented to us evidence, by practitioners other than Dr. Schafer, that his method had given good results, and until so many observers had expressed this view that we felt we had no right, in view of our own lack of experience, to condemn the plan. Fully appreciating that fact that Dr. Schafer was an unknown factor in work along these lines and that his views seemed antagonistic to these generally held by the profession regarding antitoxic substances and vaccines, we appended under the title of his paper an editorial statement in which we expressed our recognition of the rather revolutionary character of the views which he advanced, the idea being that we held ourselves in no way responsible as indorsers of his opinion, but presented his statements to the profession for what they might be worth. We confess, too, that for many months after publishing his article we could not bring ourselves to the employment of the products which he advocated for the reasons that we have just given; but as time went by other practitioners reported such extraordinary good results, by word of mouth and otherwise, that we could not fail to be impressed with the fact that the cures produced were not the result of mistaken observation or enthusiasm, and finally we employed some of these substances ourselves. We are free to admit that we have not cured every case by this means, but there can be no doubt that in some instances the results which have ensued have been no less than remarkable. Furthermore, we know of cases, notably gonorrhoeal rheumatism so-called in which this method of treatment has produced a cure in an astonishingly short period of time when every other known remedy, including antigonococcic serum, has failed.

We do not believe that there will ever be a remedy evolved by the ingenuity of man which will be capable of renovating, so to speak, tissues which have been gravely damaged or totally destroyed by the fire of an active infection, any more than we believe that any one will ever be skilful enough to devise a substance which will make a decayed apple a good apple, or any more than we believe that diphtheria antitoxin or tetanus antitoxin can ever be made so efficient that after a cell has been irretrievably damaged by disease it can completely recover all of its functions. Neither do we believe in condemning any remedy reported to be efficacious by a multitude of our colleagues, even if we cannot explain how it does good.

Recently Schafer's so-called phylacogens have been vehemently attacked, but no evidence that this attack is based on experience has been adduced. The attack seem to be based upon the theories which animated us when we appended the foot-note to Dr. Schafer's article at the time we originally published it, and the difference between our attitude then and now is that we have seen results which have forced us to cast theory aside and accept fact.

The object of the editors of the Therapeutic Gazette in the twenty-two years in which it has been under their control, and during which time they have been absolutely untrammelled in its control by any influence whatever, has been to present to the profession facts which would be useful to it in its efforts to relieve humanity and practice medicine successfully. We have endeavored whenever it was possible, in everything we have done, to make the practice of therapeutics rational, and to call attention to those advances in physiology, pathology, and bacteriology which explained results hitherto obtained by empirical methods. We hold no brief for any product, but there are two briefs that we hold tenaciously: The first is that we recognize that in the struggle with disease the physician should neither embrace nor condemn any measure without giving it careful consideration and endeavoring to clearly understand exactly how it can do good. The second brief is that we have no hesitation in calling the attention of members of the profession to products, when there seems to be undeniable evidence that these products do good, even if it is not as yet clear how they do good.

If an individual, or any set of individuals, by fortuitous circumstances, good luck, or research, discovers a remedy which does good in conditions in which other remedies fail, we believe that this product should be employed by the profession, not only in order that it may determine for itself how good results are obtained, but because the profession recognizes that its most sacred duty is to do the best thing for the patient.

The writer of this editorial for many years has endeavored to explain empirical practice by the results of scientific research, has constantly endeavored to carry on this work and to do all he can to make our measures rational, but no one who piactices medicine actively can fail to appreciate the fact that if we used only those remedies in which accurate scientific observations tell us how they act, we would get but a small proportion of the good results which now accure. For many years quinine was used with no scientific basis whatever. Mercury, which notwithstanding the advent of salvarsan still has a wider sphere of usefulness than that drug, is still unexplained as to its effects in syphilis, and so are the effects of the iodides. Innumerable instances of a similar nature may be cited, as, for example, the salicylates in the rheumatic diathesis and in acute articular rheumatism.

Recently a widely read journal has asserted that physicians "have no moral right to employ remedies of whose nature they are ignorant." If by this term "nature" the origin and composition of the substance are meant, it may be possible to hold that this view is correct; but if by this term it is meant that when we have no knowledge as to how a remedy does good we should discard it, we believe it is incorrect, and if the words quoted are true, ninety-nine per cent. of the profession ignore them, because they recognize that where scientific explanation for the action of a remedy is lacking, and clinical experience indicating its usefulness is present, it is their duty to resort to such a remedy, hoping, and striving, to the end that it may be placed upon a rational basis.

As Hervey well says in a recent issue of the New York Medical Journal: "The practice of medicine is replete with problems which depend for solution, not upon exact experiment, but upon the judgment of experience." In other words, we are forced, by lack of scientific knowledge, to use many remedial agents because they have been found curative, and every time they are employed a more accurate conception of their exact value and manner of action is approached until finally we discover their exact value. If the phylacogens are worthless, as some criticisms based on theory assert, they will soon go into the limbo of forgotten things; but if they do not do harm and often do great good, they will be more and more employed, because our first duty is to cure if it be posisble. At present all the facts at hand point to the view that they are not cure-alls but very valuable in many cases, and until the present evidence is refuted by experience they must be given fair trial and not condemned off-hand, the more so as trial so far gives good results in suitable cases and only theory condemns.—*Editorial Therapeutic Gazette, March* 1913.

---

Many a distressing frontal headache may be relieved by reducing the hypertrophy of a middle turbinate, preferably by streaking with trichloracetic acid.—*American Journal of Surgery.*

